# Protective effects of blocking PD-1 pathway on retinal ganglion cells in a mouse model of chronic ocular hypertension

**DOI:** 10.3389/fimmu.2022.1094132

**Published:** 2023-01-18

**Authors:** Siqi Sheng, Yixian Ma, Yue Zou, Fangyuan Hu, Ling Chen

**Affiliations:** ^1^ Department of Ophthalmology & Vision Science, Eye & Ears, Nose and Throat (ENT) Hospital, Shanghai Medical School, Fudan University, Shanghai, China; ^2^ Key National Health Coucil (NHC) Key Laboratory of Myopia, Fudan University, Shanghai, China; ^3^ Laboratory of Myopia, Chinese Academy of Medical Sciences, Shanghai, China; ^4^ Shanghai Key Laboratory of Visual Impairment and Restoration, Eye & Ears, Nose and Throat (ENT) Hospital, Shanghai Medical School, Fudan University, Shanghai, China

**Keywords:** glaucoma, retinal ganglion cell, microglia polarization, programmed cell death 1 ligand 1, programmed cell death 1 ligand 2, neuroinflammation, neuroprotection, apoptosis

## Abstract

**Purpose:**

In this study, we aimed to investigate whether Programmed cell death 1 ligand 1/programmed cell death 1 ligand 2 (PD-L1/PD-L2) double knockout (dKO) has a protective effect on RGCs in a mouse model of chronic ocular hypertension (COHT).

**Methods:**

We used superparamagnetic iron oxide to induce COHT in mice. Apoptosis of retinal ganglion cells (RGCs) and activation of microglia were evaluated using western blotting (WB) and immunofluorescence staining of the mouse retina. In addition, we also conducted transcriptome sequencing and further gene expression analyses using the gene ontology (GO) and Kyoto Encyclopedia of Genes (KEGG) database.

**Results:**

In the mouse model of COHT, PD-L1/PD-L2 prevented the apoptosis of RGCs to some extent. Blocking the programmed cell death 1 (PD-1) pathway also increased the number of anti-inflammatory M2-activated microglia and enhanced the phosphorylation of its related pathway signal transducer and activator of transcription (STAT)6. Sequencing results showed that this protective effect may have been achieved by regulating the NF−B, tumour necrosis factor (TNF), PI3K/Akt and toll-like receptor signaling pathway etc.

**Conclusion:**

Blocking the PD-1 pathway has a protective effect on RGCs in the mouse model of COHT induced by superparamagnetic iron oxide.

## Introduction

Glaucoma is the most common optic neuropathy, which is characterised by the apoptotic death of the retinal ganglion cell (RGC) somata, resulting in irreversible loss of vision (1–3). Although the exact molecular mechanism of RGC apoptosis is unclear, growing evidence shows that microglial activation and neuroinflammation exert a central function in the early and late stages of glaucoma-associated neurodegeneration (4–7). In human and experimental models of glaucoma, activated microglia can be identified in the optic nerve papilla and retina (8–13), and the degree of microglial activation is correlated with the degree of neurodegeneration (14, 15). In addition, minocycline (5, 8) or antibodies directed against tumour necrosis factor-alpha (TNF-α) (10) can be used to block microglial activation (11) in experimental glaucoma models, prevent immune cell infiltration, and significantly reduce RGC death. The obtained data indicate that activated microglia are the driving force of glaucoma-induced neurodegeneration. Nevertheless, the molecular mechanism mediating microglial activation in glaucoma remains unclear (12).

Activated microglial cells have distinct differentiation types, namely M1 and M2. Activated M1 microglia triggers the signal transducer and activator of transcription (STAT)1 signalling pathway leading to high levels of pro-inflammatory factors, including interleukin (IL)-12, IL-1β, and TNF-α. Activated M2 microglia initiates the STAT6 signalling pathway which stimulates the release of anti-inflammatory factors, transforming various neurotrophic factors, such as growth factor βand IL-10 (13). Selectively inhibiting the overactivation of microglial cells and limiting their differentiation into the M1 type while retaining neuroprotective, anti-inflammatory, and nerve regeneration-promoting functions of the M2 microglia may be an ideal method for the treatment of neurodegenerative diseases.

Programmed cell death 1 (PD-1), a vital cellular regulatory molecule in the immune system (14), is primarily expressed in activated T and B lymphocytes (15, 16). There are two known ligands of PD-1—programmed cell death 1 ligand 1 (PD-L1) and programmed cell death 1 ligand 2 (PD-L2). PD-L1 is widely distributed in the lung, heart, kidney, spleen, and central nervous system. PD-L2 expression is restricted to antigen-presenting cells, such as dendritic cells, macrophages, and microglia. By binding with its ligands, PD-1 regulates the activation and inactivation of T cells and inhibits abnormal T cell activation responses (14, 17). Our previous studies found that double knockout (dKO) of the PD-L1/PD-L2 genes in mice can increase the number of RGCs (18) and reduce the number of retinal microglia, while the number of M2-activated microglia is increased and the STAT6 pathway is activated accordingly.

We speculate that after the injury of RGCs, the abnormally expressed PD-1 molecule on the RGCs (19) combines with the ligand PD-L1/PD-L2 on the surface of microglia cells to start the immune regulation mechanism of the interaction between microglia and RGCs, thus affecting the fate of RGCs. In this study, we used superparamagnetic iron oxide to induce chronic ocular hypertension (COHT) in PD-L1/PD-L2 dKO mice. RGC apoptosis and microglial activation were assessed by employing western blot (WB) and immunofluorescence staining techniques for the retina. Using this model, we also explored the function of the PD-1 signalling system in controlling the microglial activation type to identify important target molecules for the treatment of glaucoma-induced RGC injury.

## Materials and methods

### Animals

This study obtained mice, including wild-type (WT) C57BL/6J, Cd274[-/-], and Pdcd1lg2[-/-] mice, from Cyagen Biosciences Inc. (Suzhou, China). Programmed death ligand 1 (PD-L1) is encoded by Cd274 gene, and programmed death ligand 2 (PD-L2) is encoded by Pdcd1lg2 gene.The mouse Cd274 gene (GenBank accession number: NM_021893.3; Ensembl: ENSMUSG00000016496) is located on mouse chromosome 19. 7 exons have been identified, with the ATG start codon in exon 2 and TAA stop codon in exon 7. The mouse Pdcd1lg2 gene (GenBank accession number: NM_021396.2; Ensembl: ENSMUSG00000016498) is located on mouse chromosome 19. 6 exons have been identified, with the ATG start codon in exon 2 and TAG stop codon in exon 5. Exon 3 of mouse Cd274 to exon 5 of mouse Pdcd1lg2 were selected as target region. Cas9 and gRNA were co-injected into fertilized eggs for knockout (KO) mouse production. The pups were genotyped by PCR followed by sequence analysis. All experiments were carried out following guides from the Animal Care and Use Committee of Fudan University (Shanghai, China). Our study protocols were approved by the Association for Research in Vision and Ophthalmology Statement for the use of animals in ophthalmic and vision research. To avoid errors caused by sex differences, the same number of male and female mice were included in each experimental group.

### Superparamagnetic iron oxide-induced model of elevated intraocular pressure

Mice were anaesthetised using an intraperitoneal injection of chloral hydrate (500 mg/kg). An increase in IOP was triggered unilaterally by the injection of 2 μL superparamagnetic iron oxide (Bang Laboratories, Inc.) into the anterior chamber of the right eye of each animal based on a surgical microscope according to previous report (20, 21). Mice that developed signs of inflammation (including clouding of the cornea, oedematous cornea, iris prolapse, lens injury, and hyphema) were excluded from the study.

### IOP measurements

IOP of mice was measured on the 3rd, 7th, 10th, 14th, 20th and 30th days after superparamagnetic iron oxide injection. The measurement of IOP was made in conscious mice with a hand-held rebound tonometer (Icare TonoLab; Colonial Medical Supply, Franconia, NH). In brief, the tonometer was positioned in front of the eye, and thus the probe hit the cornea perpendicularly. After eliminating the highest and lowest values, the average IOP was presented automatically when six measurements were made. In addition, machine-generated mean was regarded as one reading. Three measurements were made at a given time point. At the same time, the average of these three values was recorded as the overall IOP. Because the IOP varies throughout the day, all IOPs were made at the same time of day (between 14:00 and 17:00 h).

### Haematoxylin and eosin staining

Eyeball specimens were fixed in formalin the night before and were embedded and sectioned into 10-μm slices. Before staining, deparaffinisation was performed by applying xylene. Rehydration of sections was performed in an ethanol gradient before washing in a phosphate-buffered solution (PBS). Subsequently, the sections were stained with HE. A microscope (Leica DM4000, Germany) was employed to analyse retinal tissue morphology.

### Western blot

After lysis of mouse retinas in the RIPA buffer (Beyotime, Shanghai, China), the BCA approach (Beyotime) was utilised to determine protein content. Proteins (around 20 μg) were separated through 10% or 12% SDS-PAGE, followed by transfer on PVDF membranes (0.45 μm; Millipore, Shanghai, China). Later, 5% Bovine Serum Albumin was utilised to block membranes under ambient air for a 1-h period, followed by overnight incubation under 4°C using anti-Iba1 (1:1000, #ab178846, Abcam, Cambridge, MA, USA), anti-iNOS (1:1000, #ab178945, Abcam, Cambridge, MA, USA), anti-CD206 (1:200, AF2535, R&D Systems), anti-STAT1 (1:1000, #ab239360, Abcam, Cambridge, MA, USA), anti-STAT1 (phospho S727, 1:1000, #ab109461, Abcam, Cambridge, MA, USA), anti-STAT6 (1:1000, #ab32520, Abcam, Cambridge, MA, USA), and anti-STAT6 (phospho Y641, 1:1000, #ab263947, Abcam, Cambridge, MA, USA), with β-Tubulin being the endogenous reference. Membranes were rinsed sufficiently through PBST three times. Subsequently, the membrane was exposed to 1-h HRP-labelled secondary antibody incubation. At last, chemiluminescence was used to analyse the expression of each protein.

### Immunofluorescence staining

After the eye specimens were rapidly obtained from anaesthetised mice with no perfusion fixation, they were subject to 1-h fixation using 4% paraformaldehyde (PFA) under 4°C, followed by the removal of the anterior segment of the eyes. Later, the specimens were dehydrated using gradient sucrose solutions (10% for 30 min and 20% for overnight dehydration) under 4°C, followed by embedding of eyecups within the optical coherence tomography (OCT) compound (Tissue Tek, Torrance, CA, USA). Thereafter, this assay sliced the retina into 10-μm vertical sections with a freezing microtome (Leica, Nussloch, Germany). The retinal slices were rewarmed at room temperature for 30 min, followed by 15-min 4% PFA fixation, 1-h blocking using the solution that contained 0.1% Triton X-100 and 5% goat serum albumin supplemented within PBS under ambient temperature for a 1-h period, and overnight probing using primary antibodies under 4°C. Primary antibodies listed below were used in this study, rabbit-anti-Iba1 (1:100, #ab178846, Abcam, Cambridge, MA, USA), goat-anti-CD206 (1:8, #AF2535, R&D Systems), and rat-anti-CD16/CD32 (1:50, #BD553141, BD Biosciences). Membranes were then sufficiently rinsed using PBS, followed by an additional 1-h fluorescence secondary antibody incubation (1:1000), anti-rat IgG Alexa-Fluor-555 (Thermo Fisher Scientific, Inc., USA), anti-rabbit IgG Alexa-Fluor-488 (Thermo Fisher Scientific, Inc., USA), and anti-goat IgG Alexa-Fluor-633 (Thermo Fisher Scientific, Inc., USA) under ambient temperature. Sections and coverslips were washed and sealed using the anti-fade mounting agent and stained with 4’,6-diamidino-2-phenylindole (DAPI) (Abcam, Cambridge, MA, USA). This work employed Olympus FV1000 confocal laser scanning microscope for image acquisition. The value of Iba1, CD16/CD32, and CD206 positive cells were calculated. Image-Pro Plus software (version 6.0; Media Cybernetics Inc., Rockville, MD, USA) was used to analyse immunofluorescence images.

### RNA isolation and library preparation

TRIzol reagent was used to extract total RNA with the manufacturer’s instructions. The NanoDrop 2000 spectrophotometer (Thermo Fisher Scientific, USA) was used for evaluating RNA content and quality. Agilent 2100 Bioanalyzer (Agilent Technologies, Santa Clara, CA, USA) was used for assessing RNA integrity. TruSeq Stranded mRNA LT Sample Prep Kit (Illumina, San Diego, CA, USA) was used in library construction following specific protocols. OE Biotech Co. Ltd. (Shanghai, China) is credited for transcriptome sequencing and analyses.

### RNA sequencing and differentially expressed genes analysis

This study applied Illumina HiSeq X Ten platform for library sequencing to produce paired-end reads of 150 bp. First, Trimmomatic was used to process raw reads in the FASTQ format (22), while clean reads were obtained by removing low-quality ones. Later, HISAT2 was utilised to map clean reads against human genome (GRCh38) (23). Cufflinks was utilised to determine gene FPKM (24, 25), whereas HTSeqcount (26) was utilised to obtain the gene read numbers. R package DESeq (2012) was used to analyse DEGs (27) selected upon the thresholds of Foldchange (FC)>2 or <0.5 and p<0.05. DEGs expression profiles within diverse samples and groups were analysed by hierarchical clustering. According to hypergeometric distribution, DEGs were then subject to gene ontology (GO) as well as Kyoto Encyclopaedia of Genes and Genomes (KEGG) (28) analysis with R package. StringTie was subsequently applied in read reassembly (29), and reference genome was compared against known genes annotated to extend gene structure and identify new transcripts with Cuffcompare. The number of gene counts in each sample was standardised using the DESeq2 software, and the expression was assessed as the base mean value. The difference multiple was calculated, and the significance level of the difference was evaluated using a negative binomial distribution test. Finally, protein-coding DEG were screened in accordance with the results of the difference multiple and different significance test. After identification, DEGs were enriched and analysed by GO. DEG functions were described and combined with GO annotation results. In the GO function enrichment analysis, the numbers of DEGs of each GO entry were counted, and the significance of DEG enrichment of each GO entry was computed with the use of a hypergeometric distribution algorithm. The calculation returned a P-value describing enrichment significance (each biological process, cellular component, and molecular function was computed using Fisher’s exact test). The lower the P-value, the greater the statistical significance. Genes for follow-up studies were selected according to GO analysis results and biological significance. KEGG is the main public database used for pathway analysis of differentially expressed protein-coding genes, and the significance of DEG enrichment in each pathway was computed by adopting a hypergeometric distribution test.

### Statistical analysis

Data were analysed by GraphPad Prism (ver. 9.0, GraphPad Software, USA). Quantitative results were presented as mean ± SD. Two groups were compared using t-test (two-tailed) and Fisher’s exact test. P<0.05 was considered statistically significant.

## Results

### Injection of superparamagnetic iron oxide into the anterior chamber increases the IOP of WT and PD-L1/PD-L2 dKO mice

**Figure 1:**
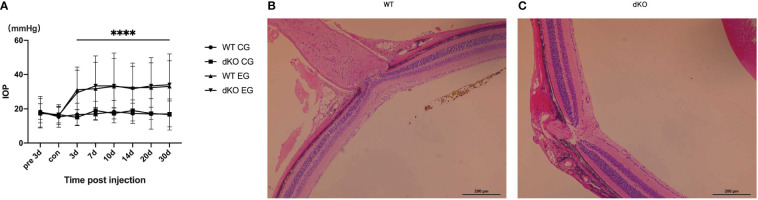
Intraocular pressure (IOP) detection in mice. **(A)** IOP values measured using rebound tonometry in wild-type (WT) and programmed cell death 1 ligand (PD-L)1/PD-L2 double knockout (dKO) mice injected with superparamagnetic iron oxide. Data are indicated as the mean IOP ± standard deviation (SD), n=6 mice per group. IOP values were obviously different on days 3–30 in WT and dKO mice receiving microsuperparamagnetic iron oxide in relative to WT and dKO control mice without injection (****P<0.0001). CG: Control Group, which means control mice without injection. EG: Experimental Group, which means experimental mice receiving microsuperparamagnetic iron oxide injection. **(B, C)** Representative images of hematoxylin and eosin (HE) staining of the central retina near the optic nerve in WT **(B)** and dKO mice **(C)** 30 days after superparamagnetic iron oxide injection. Data are indicated as the mean ± SD; t-test (n=6 per group).

Injection of superparamagnetic iron oxide superparamagnetic Iiron oxide into the anterior chamber of mice to block aqueous humour outflow and simulate angle-closure glaucoma (20, 30) increased the IOP in both WT and PD-L1/PD-L2 dKO mice (**Figure 1A**). Each mouse received a superparamagnetic iron oxide injection into the right eye, the experimental eye, whereas the left eye, the control eye, did not receive such injections. IOP monitoring began 3 days before the administration of the superparamagnetic iron oxide injection. The IOP values of WT or PD-L1/PD-L2 dKO mice that did not receive superparamagnetic iron oxide injections were not significantly different at any time point, whereas the IOP values of experimental eyes significantly increased from day 3 to day 30 after the superparamagnetic iron oxide injection was administered. The IOP values were not significantly different between WT and PD-L1/PD-L2 dKO mice (**Figure 1A**). Haematoxylin and eosin (HE) staining of the experimental eyes 30 days after the administration of the superparamagnetic iron oxide injection showed no significant difference in retinal morphology between WT (**Figure 1B**) and PD-L1/PD-L2 dKO mice (**Figure 1C**).

Data suggest that the injection of superparamagnetic iron oxide into the anterior chamber comparably increases the IOP values of WT and PD-L1/PD-L2 dKO mice.

### In the COHT model, PD-L1/PD-L2 dKO protects RGCs

**Figure 2:**
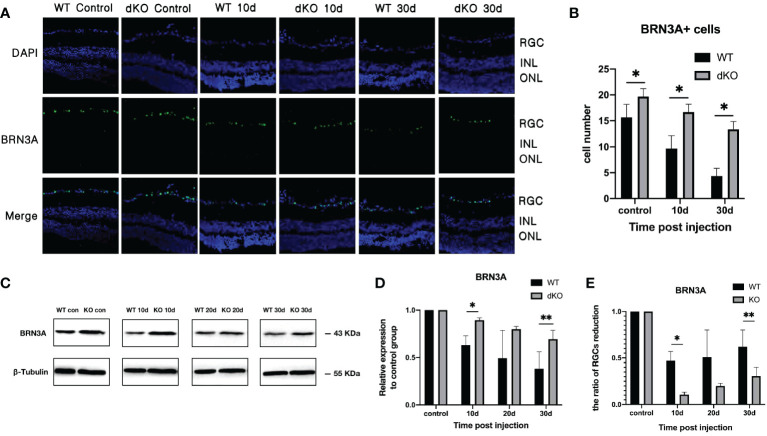
Quantification of the retinal ganglion cell (RGC) marker BRN3A in double knockout (dKO) and wild-type (WT) mice in the chronic ocular hypertension (COHT) model. **(A)** Representative immunofluorescence staining of BRN3A (green fluorescence) in the ganglion cell layer of programmed cell death 1 ligand (PD-L)1/PD-L2 dKO mice and WT mice without superparamagnetic iron oxide injection, as well as 10 and 30 days after superparamagnetic iron oxide injection. Blue fluorescence represents nuclear 4’,6-diamidino-2-phenylindole (DAPI) counterstaining. **(B)** Number of BRN3A-positive cells in immunofluorescence staining of the abovementioned region. **(C)** Western Blot (WB) was adopted for analysing the protein expression levels of BRN3A in retinal tissue lysates (10 μg) from PD-L1/PD-L2 dKO mice and WT mice 10, 20, and 30 days after superparamagnetic iron oxide injection or without superparamagnetic iron oxide injection. **(D)** The relative BRN3A content in each group of WT or dKO mice with superparamagnetic iron oxide injection was determined as the ratio of its BRN3A grey value to that of the corresponding group of WT or dKO mice without superparamagnetic iron oxide injection. β-tubulin was applied as a loading control. Data can be indicated as the mean ± standard deviation; **(E)** Calculating and comparing the ratio of RGCs reduction in WT and dKO mice for each time point after superparamagnetic iron oxide injection according to Figure 2D. t-test (dKO: n=6, WT: n=6). *P < 0.05, **P < 0.01.

BRN3A antibody can specifically tag RGCs. The effects of PD-L1/PD-L2 dKO on RGC apoptosis in the established COHT model were assessed using WB and immunofluorescence staining (31). Immunofluorescence staining of retinal sections from normal mice not exposed to superparamagnetic iron oxide injections showed large numbers of BRN3A-positive cells, indicating high numbers of RGCs in the mouse retina under normal conditions. The number of RGCs in PD-L1/PD-L2 dKO mice (19.7 ± 2.3) was higher than that in WT mice (16.1 ± 3.9), which is statistically significant. After the administration of the superparamagnetic iron oxide injection into the anterior chamber, the number of RGCs decreased with time. The number of surviving RGCs in PD-L1/PD-L2 dKO mice was greater than in WT mice on day 10 (16.8 ± 3.3) and day 30 (13.2 ± 1.5) after injection (**Figures 2A, B**). This finding was verified using WB, and substantial RGC apoptosis was detected in eyes exposed to COHT (**Figure 2C**). However, the decrease in BRN3A expression in dKO mice was lesser than that in WT mice. The BRN3A expression levels in WT mice on days 10, 20, and 30 after superparamagnetic iron oxide injection were 0.63 ± 0.11, 0.49 ± 0.38, and 0.38 ± 0.26, respectively. The corresponding values in dKO mice were 0.89 ± 0.03, 0.80 ± 0.05, and 0.68 ± 0.16, respectively (**Figure 2D**). Therefore, we calculate and compared the ratio of RGCs reduction in WT and dKO mice for each time point after superparamagnetic iron oxide injection (**Figure 2E**). On days 10, 20, and 30, RGCs reduction in WT mice are higher than those in dKO mice.

These results indicate that in this mouse COHT model, PD-L1/PD-L2 dKO prevents damage to RGCs caused by increased IOP.

### PD-L1/PD-L2 dKO reduces the number of retinal microglia and polarises them into the M2 type in the mouse model of COHT

**Figure 3:**
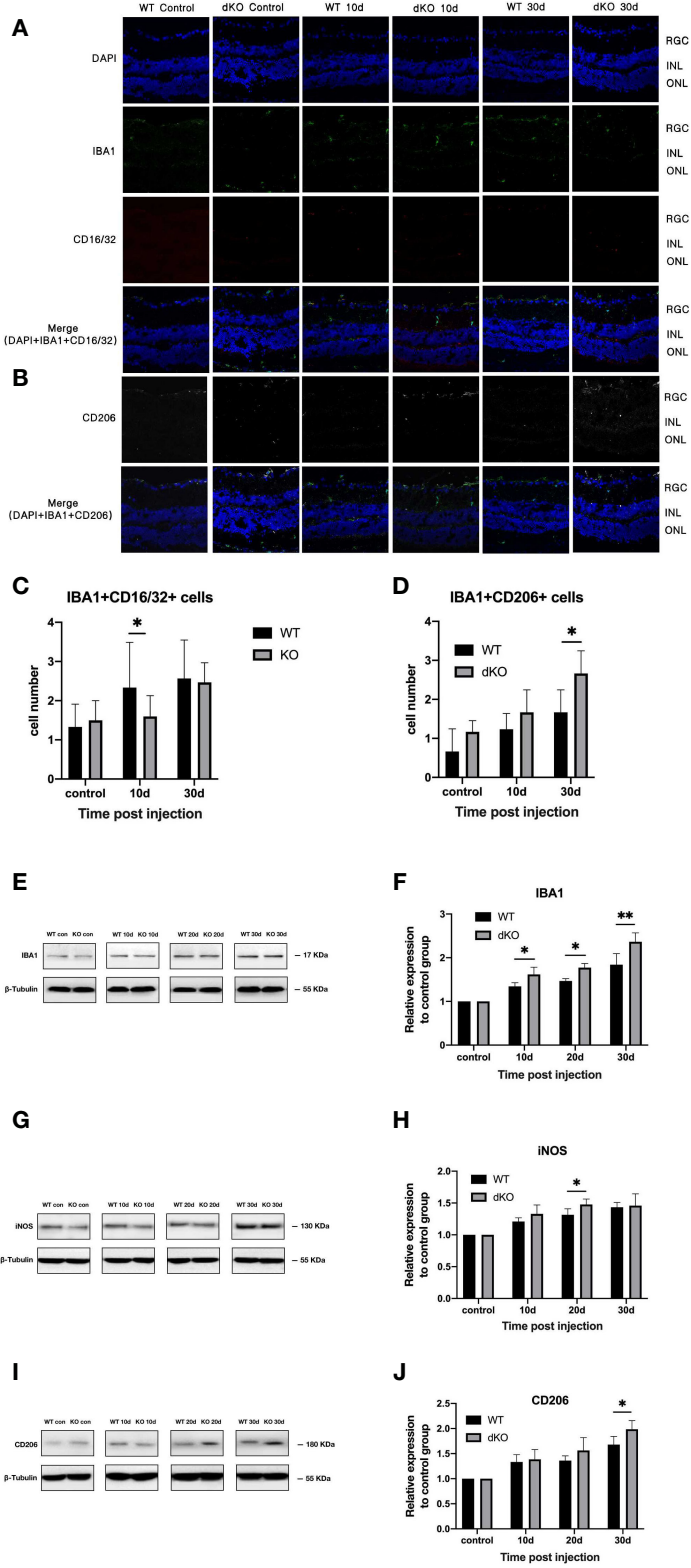
Quantification of microglial cell markers in programmed cell death 1 ligand (PD-L)1/PD-L2 double knockout (dKO) and wild-type (WT) mice in the chronic ocular hypertension (COHT) model. **(A, B)** Representative immunofluorescence staining of Iba1 (green fluorescence), CD16/32 (red fluorescence), and CD206 (grey fluorescence) in samples from PD-L1/PD-L2 dKO mice **(A)** and WT mice **(B)**. Blue fluorescence represents nuclear 4’,6-diamidino-2-phenylindole (DAPI) counterstaining. [c-d] Numbers of double-positive cells in CD16/32+Iba1+ **(C)** and CD206+Iba1+ **(D)** immunofluorescence staining in the abovementioned region. **(E–J)** Protein expression levels of the microglial cell marker Iba1 **(E)**, M1 marker iNOS **(G)**, and M2 marker CD206 **(I)** in retinal tissue lysate of PD-L1/PD-L2 dKO mice and WT mice 10, 20, and 30 days after superparamagnetic iron oxide injection or without superparamagnetic iron oxide injection. Normalised relative protein levels of Iba1 **(F)**, iNOS **(H)**, and CD206 **(J)** were calculated from the densitometric analysis of the bands. The relative protein content was determined as the ratio of the grey value of samples from WT mice in each superparamagnetic iron oxide injection group to that of samples from WT mice without superparamagnetic iron oxide injection. Similarly, the value of each time point after superparamagnetic iron oxide injection is standardised with reference to its corresponding value in the non-injected group. β-tubulin was used as loading control. Data are presented as the mean ± standard deviation; t-test (n=6 per group). *P < 0.05, **P < 0.01.

Immunofluorescence staining of microglia show that a small number of positive cell markers, such as Iba1, can be found in both WT and PD-L1/PD-L2 dKO mice without superparamagnetic iron oxide injection, and activated microglia were rarely detected. After injecting superparamagnetic iron oxide into the anterior chamber of the eye, retinal staining revealed a large number of positive cells that had migrated to the ganglion cell layer, indicating that after RGC injury, microglia were overactivated and aggregated (5–7, 32–34), accompanied by a severe inflammatory reaction. However, the retinas of WT and PD-L1/PD-L2 dKO mice showed different trends in microglial polarisation after the increase of IOP induced by the superparamagnetic iron oxide injection. Microglia activated in WT mice mainly associated with the M1 phenotype (**Figure 3A**), whereas microglia in PD-L1/PD-L2 dKO mice were mainly associated with the M2 phenotype (**Figure 3B**). The analysis of immunofluorescence-positive cells using ImageJ software showed that in animals without superparamagnetic iron oxide injection, the numbers of M1 microglial cells were almost the same in the eyes of WT mice (1.31 ± 0.64) and PD-L1/PD-L2 dKO mice (1.48 ± 0.60). After superparamagnetic iron oxide injection, the number of M1 microglia in the retina increased with time. The greatest difference between WT mice (2.32 ± 1.24) and PD-L1/PD-L2 dKO mice (1.62 ± 0.59) was observed after 10 days. After 30 days, M1-polarised microglia in WT and PD-L1/PD-L2 dKO mice were at 2.56 ± 1.12 and 2.47 ± 0.54, respectively (**Figure 3C**). M2 microglial cells in WT and PD-L1/PD-L2 dKO mice without superparamagnetic iron oxide injection were at 0.66 ± 0.61 and 1.18 ± 0.32, respectively. Retinal microglia with M2 phenotype also increased with time after superparamagnetic iron oxide injection. M2 microglia in WT and PD-L1/PD-L2 dKO mice were at 1.24 ± 0.47 and 1.67 ± 0.64, respectively, on day 10. The difference between WT (1.62 ± 0.62) and PD-L1/PD-L2 dKO (2.71 ± 0.73) mice in M2 microglia was greatest after 30 days (**Figure 3D**). WB confirmed these observations (**Figure 3G–J**) and revealed that the increase in Iba1 expression in dKO mice was greater than that in WT mice (**Figure 3E**). The Iba1 expression levels of WT and dKO mice 10 days after superparamagnetic iron oxide injection were 1.34 ± 0.09 and 1.61 ± 0.23 of those of the non-injected groups, respectively, 1.47 ± 0.05 and 1.77 ± 0.12 on day 20, and 1.84 ± 0.36 and 2.36 ± 0.30 on day 30 (**Figure 3F**).

This suggests that PD-L1/PD-L2 dKO can polarise mouse retinal microglia into the M2 phenotype.

### Effect of blocking PD-1 pathway on STAT1 and STAT6 phosphorylation in retina of mice with chronic ocular hypertension

**Figure 4:**
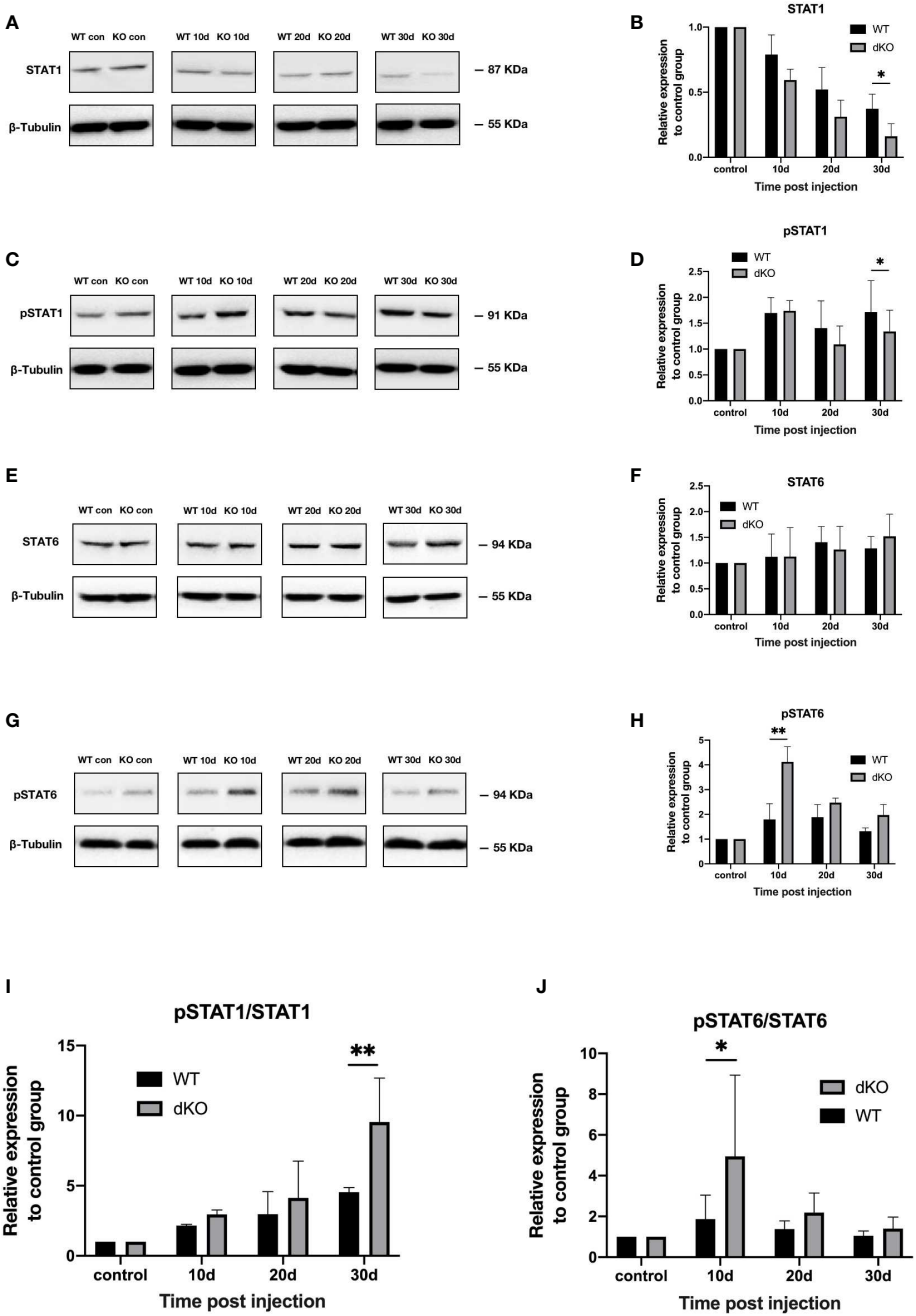
Activation of the signal transducer and activator of transcription (STAT)1 and STAT6 pathways in programmed cell death 1 ligand (PD-L)1/PD-L2 double knockout (dKO) and wild-type (WT) mice in the chronic ocular hypertension (COHT) model. [**A-H**] WB was adopted for analysing the protein expression levels of STAT1 **(A)**, pSTAT1 **(C)**, STAT6 **(E)**, and pSTAT6 **(G)** in retinal tissue lysates (10 μg) from PD-L1/PD-L2 dKO mice and WT mice 10, 20, and 30 days after superparamagnetic iron oxide injection or without superparamagnetic iron oxide injection. Bar charts comparing the average relative densities of bands of STAT1 **(B)**, pSTAT1 **(D)**, STAT6 **(F)**, and pSTAT6 **(H)** expression in retinas of PD-L1/PD-L2 dKO and WT mice. The relative maker content in each group of WT or dKO mice with superparamagnetic iron oxide injection was determined as the ratio of its marker grey value to that of the corresponding group of WT or dKO mice without superparamagnetic iron oxide injection. β-tubulin was applied as a loading control. Data can be indicated as the mean ± standard deviation; **(I, J)** The ratios of pSTAT1 to STAT1 **(I)** and pSTAT6 to STAT6 **(J)** represent the degree of STAT1 and STAT6 activation, respectively. In all bar charts, each time point after superparamagnetic iron oxide injection is standardised with reference to its corresponding value in the non-injected group (n=6 per group). *P<0.05 and **P<0.01 vs. the data; t-test.

In general, STAT1 phosphorylation is increased in M1-activated microglia, whereas STAT6 phosphorylation is upregulated in M2-activated microglia (35). **Figure 4** shows that following superparamagnetic iron oxide injection, STAT1 phosphorylation levels in the retinas of WT mice and dKO mice increased, representing an increased activation of M1 microglia. The degree of STAT1 phosphorylation in dKO mice was greater than that in WT mice on days 10 (2.95 ± 0.32 and 2.16 ± 0.14), 20 (4.14 ± 2.76 and 2.97 ± 1.71), and 30 (9.54 ± 3.55 and 4.54 ± 0.38) after superparamagnetic iron oxide injection (**Figure 4I**). For STAT6, the phosphorylation levels of dKO mice were greater than that of WT mice on days 10, 20, and 30 after injection. The values were 4.95 ± 4.49 vs. 1.87 ± 1.43, 2.19 ± 1.13 vs. 1.38 ± 0.35, and 1.40 ± 0.69 vs. 1.01 ± 0.17, respectively (**Figure 4J**). Our study found that the phosphorylation degree of STAT1 and STAT6 in both WT and dKO mice increased after receiving superparamagnetic iron oxide injection, especially in dKO mice. The difference was that the phosphorylation degree of STAT1 increased over time, while the phosphorylation degree of STAT6 decreased over time. Therefore, receiving superparamagnetic iron oxide injection will enhance the polarization of M1 and M2 of mouse retinal microglia, especially in dKO mice.

So we infered that dKO mice can promote the polarization of M1 and M2 microglia in COHT model.

### Changes of mRNA expression in PD-L1/PD-L2 dKO mice in COHT model

**Figure 5:**
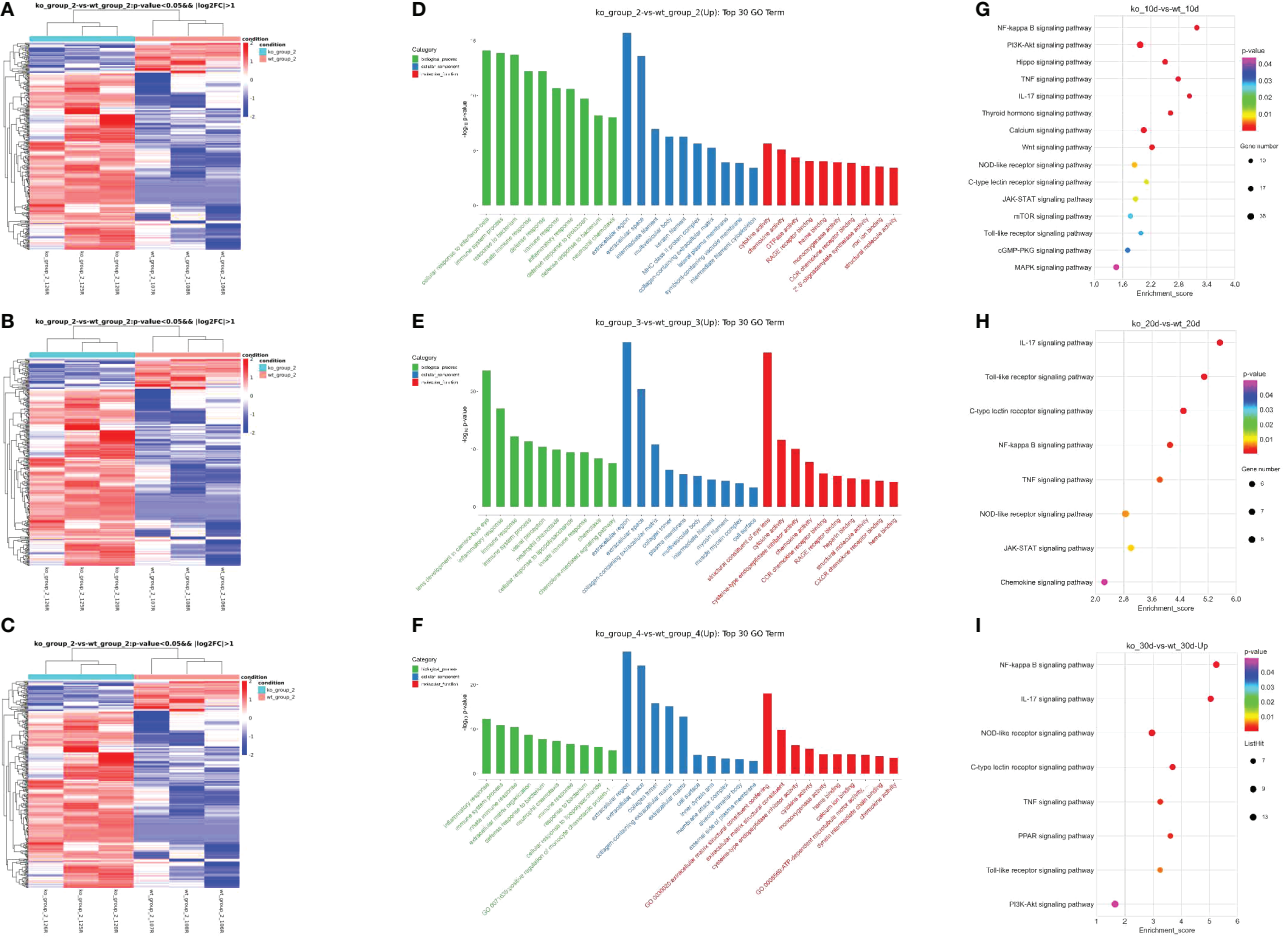
Differentially expressed genes in programmed cell death 1 ligand (PD-L)1/PD-L2 double knockout (dKO) and wild-type (WT) mice in the chronic ocular hypertension (COHT) model. **(A–C)** Heatmap graph for DEGs. Heatmap for DEGs based on 10 days **(A)** after superparamagnetic iron oxide injection between WT and dKO mice. Heatmap for DEGs based on 20 days **(B)** after superparamagnetic iron oxide injection between WT and dKO mice. Heatmap for DEGs based on 30 days **(C)** after superparamagnetic iron oxide injection between WT and dKO mice. Red represents relatively high expression protein coding genes, and blue represents relatively low expression protein coding genes. **(D–F)** GO enrichment analysis results are shown. 10 days **(D)** after superparamagnetic iron oxide injection between WT and dKO mice, 20 days **(E)** after superparamagnetic iron oxide injection between WT and dKO mice and 30 days **(F)** after superparamagnetic iron oxide injection between WT and dKO mice.The horizontal axis reveals the name of the GO entry, and the vertical axis stands for the -log10 P-value. **(G–I)** KEGG enrichment analysis results are shown. Similarly, 10 days **(G)** after superparamagnetic iron oxide injection between WT and dKO mice, 20 days **(H)** after superparamagnetic iron oxide injection between WT and dKO mice and 30 days **(I)** after superparamagnetic iron oxide injection between WT and dKO mice.The horizontal axis reveals the name of the GO entry, and the vertical axis stands for the -log10 P-value. The horizontal axis represents the enrichment score. In addition, it indicates the larger the bubble, the higher the number of differential protein-coding genes contained, and the bubble colour changes from purple, blue, green, and red. Besides, it suggests that the smaller the enrichment P-value, the greater its significance.

To explore changes of mRNA expression in PD-L1/PD-L2 dKO mice in COHT model, we investigated differential gene expression analysis between WT and dKO mice at different time poionts (10, 20 and 30 days) in the COHT model. A total of 289 DEGs were found in 10 days group including 244 up-regulated DEGs and 45 down-regulated DEGs (**Figure 5A**), the data in 20 days group were 399 DEGs in total including 327 up-regulated DEGs and 72 down-regulated DEGs (**Figure 5B**). Moreover, 1038 DEGs were obtained in 30 days group including 461 up-regulated DEGs and 577 down-regulated DEGs (**Figure 5C**).

In order to understand the gene function and the pathway of valid DEGs involved, we performed GO and KEGG analyses of 1726 DEGs. The top ten terms of biological processes (BPs), cellular component (CCs) and molecular functions (MFs) were listed separately (**Figures 5D–F**). In BPs, GO functions were mainly enriched in immune system process, innate immune response and immune response. CCs regarding extracellular region, extracellula space and collagen-containing extracellula matrix. MFs including cytokine activity and chemokine activity were found.

Similarly, the pathways related to immune processes and regulations were also found in KEGG enrichment analysis, such as the NF-κB, TNF, PI3K/Akt and toll-like receptor signalling pathways etc. (**Figures 5G–I**). These results suggested that immune factors were critical in COHT of the blocking PD-1 pathway.

## Discussion

The current study evaluates the protective effects of neuronal PD-L1 on RGCs in a mouse model of COHT induced by superparamagnetic iron oxide. During postnatal retinal maturation in mice, 50% of RGCs undergo programmed cell death, which is one of the key homeostasis mechanisms regulating cell numbers, and our previous study findings suggest that PD-1 signalling can reduce apoptosis in neonatal RGCs (19) and dKO of the PD-L1/PD-L2 genes can increase the number of RGCs in mice (18). Our current results likewise prove that in the mouse model of COHT induced by superparamagnetic iron oxide injection into the eye, dKO of the PD-L1/PD-L2 genes reduces RGC injury. This indicates that blocking the PD-1 pathway provides, to a certain extent, protection for RGCs in the mouse model of glaucoma induced by superparamagnetic iron oxide.

Although the role of neuroinflammation in human glaucoma is uncertain, a large number of evidences reveals the close relationship. Neuroinflammation is currently regarded as a double-edged sword, causing both harmful and helpful impacts on neurons (36). Numerous lines of evidence show that microglia are neurotoxic (37), whereas other data show that neuroinflammation can be beneficial by stimulating myelin repair, removing toxic aggregation proteins as well as cell debris from the central nervous system, and secreting neurotrophic factors with the purpose of preventing nerve injury (38–40). Immune cells such as microglia in the central nervous system seem to be heterogeneous, with a variety of functional phenotypes ranging from pro-inflammatory M1 phenotype to immunosuppressive M2 phenotype. Because the two microglia phenotypes can be converted into the other, microglia can be stimulated to protect neurons by modulating microglial phenotypes. For example, lipopolysaccharide (LPS) treatment enables microglial BV2 cells and primary microglia to adopt the M1 phenotype, whereas the addition of the Rho kinase inhibitor fasudil shifts their state to M2 microglia, which is characterised by low NF-κB activity, reduced levels of proinflammatory cytokines (IL-1β, IL-6, and TNF-α) together with enhanced levels of anti-inflammatory cytokines (IL-10) (41). In the LPS model, fasudil increased Arg1+/CD11b+ M2 microglia and decreased iNOS+/CD11b+ M1 microglia (41). It is shown that the inhibition of the NADPH oxidase or gene deletion of its functional p47phox subunit switches microglial activation in response to LPS from M1 to M2 (42). In addition to drug therapy, epigenetic mechanisms can also be used to regulate the phenotype of M2 microglia. Histone H3K27me3 demethylase jmjd3 has been shown to be necessary to replace microglial activation (43). Inhibition of jmjd3 can inhibit the expression of Arg1 and Cd206 in IL-4-treated microglia, enhance the production of pro-inflammatory cytokines and NO, and finally facilitate the death of dopaminergic neurons (43). In our current study, we found that PD-L1/PD-L2 dKO transformed mouse retinal microglia into the M2 phenotype, which may explain the protective effect on RGCs.

STAT1 and STAT6 are downstream molecules of M1 and M2 microglia, separately (44). Many studies have shown that STATs are key factors in the polarisation of M1 and M2 macrophages (45, 46). INF-γ released by lymphocytes upregulates pSTAT1, which is necessary to promote the activation of M1 macrophages (47). By contrast, in Th2 cell-mediated immune responses, pSTAT6 is an important regulator of M2 macrophage polarisation in the existence of IL-13 or IL-4 (48, 49). Physiolin D can also control macrophage M1/M2 polarisation *via* the STAT1/6 pathway (46). Our results in the model of COHT induced by superparamagnetic iron oxide showed that the phosphorylation level of STAT6 in PD-L1/PD-L2 dKO mice was always higher than that in WT mice. The increase of the phosphorylation level of STAT6 in PD-L1/PD-L2 dKO mice can explain the increase of M2 microglia. However, the phosphorylation degree of STAT6 in dKO mice in the COHT model decreased over time, which was inconsistent with the result that M2 microglia increased over time, because the phosphorylation level of STAT6 did not show an absolute linear correlation with M2 polarization of microglia.

Our mRNA sequencing results show that in PD-L1/PD-L2 dKO mice, PI3K/Akt signal pathway was upregulated. Research has provided part evidence that the PI3K/Akt pathway protects the retina in non-glaucoma models (50–55), and in many eye diseases, neuroprotective effects are exerted by activating the PI3K/Akt pathway to prevent RGC injury (56, 57). This may also explain the increase in the number of RGCs in PD-L1/PD-L2 dKO mice. Likewise, the NF-κB signalling cascade can regulate the production of proinflammatory mediators and make contributions to the M1/M2-like transformation of microglial cells (58, 59). Sema3A activates NF-κB by increasing Rac1 and p65 levels, enhances LPS-induced acute renal injury, and promotes LPS-induced macrophage activation as well as cytokine production through the plexin-a4-dependent manner (60, 61). Previous studies have verified that the expression of toll-like receptor 4 in the retina is increased in the optic nerve crush mouse model (62). Trif KO can inactivate the NF-κB signalling pathway and reduce the release of proinflammatory cytokines by hindering the activation of microglia in the mouse retina (63). Many other key inflammatory pathways are involved in the pathogenesis of glaucoma and are normal in human and animal models of glaucoma, for instance, toll-like receptor (64, 65) and TNF-α (10, 11, 66) pathways, among others. We speculate that blocking PD-1 involves the regulation of NF-κB and other signalling pathways that induce microglial polarisation into the M2-like phenotype. Nevertheless, the exact molecular pathway remains to be verified.

The inflammatory background in glaucoma is also inseparable from the interaction between T cells and retinal microglia. As previously mentioned, the blocking of PD-1 pathway may affect the activity of T cells at the same time, especially T helper cells and regulatory T cells. A study on anterior chamber related immune deviation found that CD4+PD-1+T cells in murine spleen may represent a large number of regulatory T cells (67). PD-L1/PD-L2 expressed on APC in the immune system and PD-1 signal pathway on T cells activate and inhibit the activation of T cells; PD-1, which is also expressed on T cells, can also activate PD-L1/PD-L2 signals on macrophages and regulate the function of macrophages. Such two-way signals form a Reciprocal Communication between cells (17). Therefore, the interaction of PD-1 pathway, T cells and retinal microglia in glaucoma is worthy of further discussion.

Considered as a leading neurodegenerative disease, views on the pathogenesis of glaucoma are constantly updating. Although research, diagnosis and treatment are mainly focused on IOP, T cell mediated immune attack and its dynamic interaction with retinal microglia have been identified as the culprit of glaucoma. Mount of data indicate that the immune-mediated attack of the neural retina is a potential pathological process behind glaucoma, which may not be related to elevated intraocular pressure. The basic research on glaucoma autoimmunity is also a new field. Many relative theories are basically derived from the discovery of other central nervous system neurodegenerative diseases (68).

Our research also needs to be improved. PD-1 receptors are expressed on a variety of retinal cell types, for instance, astrocytes, RGCs, Mueller cells, and microglia. Additional research is required to delete PD-1 receptors in specific cell types to determine which PD-1 receptor-positive cells drive neuroinflammation in glaucoma. In addition, the extent of PD-1 effects on the death of RGCs and the activation of microglia in glaucoma should be further studied.

In summary, we found that PD-1 prevented ganglion cell injury in a mouse COHT model, participated in the dynamic regulation of M1/M2-like microglia, we also found a new breakthrough point for the study of neuroimmunological mechanisms in glaucoma-induced RGC apoptosis, and identified a novel target for the neuroprotective treatment of glaucoma-related RGC injury.

## Data availability statement

The datasets presented in this study can be found inonline repositories. The names of the repository/repositories and accession number(s) can be found below: https://www.ncbi.nlm.nih.gov/genbank/, PRJCA013510.

## Ethics statement

The animal study was reviewed and approved by Ethics Committee of Fudan University Affiliated Eye, Ear, Nose and Throat Hospital.

## Author contributions

LC conceived the study and participated in its design and coordination. SS and YM conducted intraocular injections on wild-type, dKO mice, and subsequent immunoflorescence and PCR. YZ conducted the western blot and participated in study design and data analysis. FH guided the above experimentsand. SS drafted the manuscript. All authors contributed to the article and approved the submitted version.
